# Nanovesicles released by OKT3 hybridoma express fully active antibodies

**DOI:** 10.1080/14756366.2020.1852401

**Published:** 2021-01-06

**Authors:** Mariantonia Logozzi, Rossella Di Raimo, Francesca Properzi, Stefano Barca, Daniela F. Angelini, Davide Mizzoni, Mario Falchi, Luca Battistini, Stefano Fais

**Affiliations:** a Department of Oncology and Molecular Medicine, Istituto Superiore di Sanità, Rome, Italy; b Department of Cell Biology and Neurosciences, Istituto Superiore di Sanità, Rome, Italy; c FARVA – National Centre for Drug Research and Evaluation, Istituto Superiore di Sanità, Rome, Italy; d Neuroimmunology Unit, IRCCS Santa Lucia Foundation, Rome, Italy; e National AIDS Center, Istituto Superiore di Sanità, Rome, Italy

**Keywords:** Exosomes, extracellular vesicles, immunoglobulins, OKT3 hybridoma cell line

## Abstract

Recent findings have shown that nanovesicles preparations from either primary immune cells culture supernatants or plasma contain immunoglobulins, suggesting that a natural way of antibody production may be through exosome release. To verify this hypothesis, we used the OKT3 hybridoma clone, which produces a murine IgG2a monoclonal antibody used to reduce rejection in patients undergoing organ transplantation. We showed exosome-associated immunoglobulins in hybridoma supernatants, by Western blot, nanoscale flow cytometry and immunocapture-based ELISA. The OKT3-exo was also being able to trigger cytokines production in both CD4 and CD8 T cells. These results show that nanovesicles contain immunoglobulin and could be used for immunotherapy. These data could lead to a new approach to improve the effectiveness of therapeutic antibodies by exploiting their natural property to be expressed on nanovesicle membrane, that probably render them more stable and as a consequence more capable to interact with their specific ligand in the best way.

## Introduction

The coming of monoclonal antibodies into therapy has led to a real revolution. Because of their high specificity and affinity for the target molecules, monoclonal antibodies have attracted the interest of the pharmaceutical industry, representing the fastest-growing branch of therapeutic proteins and biotechnological research^1^. Many steps forward have been made so far, starting from the first *in vitro* production of monoclonal antibodies in mice^2^, to the subsequent development of chimeric anti-human and humanised antibodies to improve half-life and reduce adverse effects^3^^,^^4^. The phage display technology^5–7^ combined with the generation of murine strains expressing human variable domains^8^^,^^9^, allowed to obtain entirely humanised antibodies. Subsequent technological advances have led to the production of single chain fragment variable (scFv) antibodies^10^, more advantageous than monoclonal antibodies in tumour therapy due to their small size, high affinity for the specific target, faster penetration of tissues and faster clearance^11^^,^^12^. Moreover, studies about the immunogenicity of mAbs have led to the development of *in silico* and *in vitro* instruments able to predict the functioning of the antibodies generated before their use in the clinic^13–17^.

An important feature of antibodies is that they are present in two forms, each with different features: (i) soluble secreted immunoglobulins, which contribute to the body’s immune surveillance; (ii) membrane-bound immunoglobulins which form B-cell Receptors and are responsible for maturation, activation, and differentiation of B cells. These latter forms, expressed in membranes, could be more functional. For example, other transmembrane type II receptors such as Fas ligand, may exert different functions when expressed on either a membrane or in its soluble form^18–20^. It is conceivable that also antibodies may be more active when expressed on a plasmamembrane. In fact, it has been shown as some monoclonal antibodies are more active in triggering cellular functions (e.g. activation, proliferation and cytokine release) when immobilised on a surface^21–23^.

The great majority of cells, including immune cells, release a variety of extracellular vesicles (EVs), in turn including nanovesicles generally called exosomes. The latter form through inward budding of the endosomal membrane and are released after the fusion of the endosomal membrane with the plasma membrane^24^. They are involved in the communication between cells and their presence both *in vitro* and *in vivo* has been widely characterised^25–28^. In 1990s, after the discovery that exosomes secreted from dendritic cells and B cells can activate CD4+ and CD8+ T cell, their potential role as vaccine vehicles raised a great interest^29–31^. It is now known that exosomes are secreted from macrophages, mast cells, T cells, epithelial cells, platelets, and tumour cells and found in many body fluids^32–35^, including human plasma^27^^,^^28^^,^^36^^,^^37^. Previous observations have shown that Western Blot analysis of plasmatic exosomes purifications may contain heavy and light immunoglobulins chains in both hamster^38^ and human plasma^39^^,^^40^. However, similar results were obtained with either human breast milk^35^ or placenta^41^, suggesting that exosomes may be a natural delivery for immunoglobulins^40^^,^^42^. Moreover, exosomes may shuttle ligands for membrane receptors, such as FasL and TRAIL, that on exosomes they are fully capable to trigger the specific receptors^43^^,^^44^. Thus, it appeared conceivable to test the hypothesis that also antibodies may be delivered by exosomes and that on exosomes they are fully active. To further support this hypothesis, we used the OKT3 cell line, that is a typical hybridoma clone producing a murine monoclonal antibody IgG2a, which recognises CD3 of human T cells^45–47^. This antibody (Muromonab-CD3) was the first monoclonal antibody to be approved for clinical use since 1985 and it is still used to reduce rejection in patients with organs transplant. In our study, exosomes were isolated from hybridoma supernatants and the presence of exosome-associated immunoglobulins was investigated. In order to associate the exosome expression of immunoglobulins with a specific function, we tested the ability of these exosomes to trigger a cytokines production associated with the interaction of the OKT3 antibody with the human CD3. The results show that exosome isolated from the OKT3 hybridoma not only express the specific antibody but this antibody is fully active in triggering cytokines production by human T cells. These results suggest that the effectiveness of therapeutic antibodies could be critically improved by using exosomes as natural sources.

## Materials and methods

### Cell culture

OKT3 hybridoma cell line was firstly produced from the fusion of a murine myeloma cell line (P3x63 Ag8.U1) and murine spleen cells immunised with human T cells^45^. OKT 3 (ATCC® CRL-8001™, Manassas, VA, USA) and P3X63Ag8 (ATCC® TIB-9™, Manassas, VA, USA) were cultured in RPMI 1640 medium with 2% foetal bovine serum (FBS) and antibiotics, at 37 °C with 5% CO_2_.

Human Primary Macrophages were obtained from buffy coats of healthy donors provided by Centro Trasfusionale Universitario Azienda Policlinico Umberto I in Rome, Italy. Briefly, primary human monocytes were obtained after the separation of peripheral blood mononuclear cell (PBMCs) through a gradient Lympholyte®-H (Cedarlane, Burlington, ON, Canada) from the buffy coats of healthy donors. PBMCs were cultured in RPMI 1640 medium with 20% FBS and antibiotics, at 37 °C with 5% CO2. The macrophages were selected by adhesion to the plate. After the addition of fresh culture medium, the monocytes were allowed to differentiate into macrophages for 7–10 days.

### Exosomes purification from cell culture supernatants

After 5 days of cell cultures, supernatants were collected, and exosomes isolated as previously described^48^. Briefly, after centrifugation of cells at 300 g for 5 min, supernatants were centrifuged at 1200 g for 15 min followed by 12,000 g for 30 min. Supernatants were then filtered using a 0.22-µm filter (Millipore Corp., Bedford, MA, USA) and centrifuged at 110,000 g for 1 h in a Sorvall WX Ultracentrifuge Series (Thermo Fisher Scientific, Waltham, MA, USA) to pellet exosomes. After one wash in a large volume of phosphate-buffered saline (PBS), exosomes were resuspended in appropriate buffers for subsequent experimental analysis.

### Flow cytometry analysis of exosomes

Exosomes purified from plasma were diluted in PBS in a final volume of 50 µL. Anti-mouse CD81 phycoerythrin (PE) conjugated (clone Eat2); antimouse CD9 Alexa Fluor 647 conjugated (clone KMC8) both purchased from Becton Dickinson (Franklin Lakes, NJ, USA) and goat antimouse IgG (H + L) respectively PE or Alexa Fluor 647 conjugated (Invitrogen, Waltham, MA, USA) were added to the exosome preparation at optimal pre-titered concentrations and left for 20 min at RT. For isotype control, Alexa Fluor 647 Rat IgG2a and PE hamster IgG1 (Becton Dickinson,Franklin Lakes, NJ, USA) were used.

About 500 µL of PBS were added to samples before the acquisition on the CytoFLEX flow cytometer (Beckman Coulter, Indianapolis, IN, USA ). The cytometer was calibrated as previously described^27^.

### Sucrose gradient

According to Théry 2002^48^, OKT3-derived exosomes were suspended in 1 ml of PBS and transferred to a 30% sucrose/D2O cushion. After centrifugation at 110,000 g for 90 min, each fraction (1 ml) was transferred in a fresh tube and after a wash in PBS for 1 h, suspended in lysis buffer for western blot analysis.

### Western blot analysis

Lysates were prepared in CHAPS buffer (10 mM Tris-HCl [pH 7.4], MgCl_2_ 1 mM, EGTA 1 mM, CHAPS 0.5%, glycerol 10%, β-mercaptoethanol 5 mM, PMSF 1 mM) containing protease inhibitor cocktail. Cell lysates and exosomes were subjected to electrophoresis on SDS-polyacrylamide gels and transferred to nitrocellulose membranes (Protran Whatman, Dassel, Germany). After blocking in 5% dry milk in PBS1X, membranes were hybridised with primary antibodies: goat anti-mouse IgG HRP (Amersham Biosciences, Milan, Italy), anti-Tsg101(4A10, GeneTex, Irvine, CA, USA) and anti-GAPDH (GA1R, Santa Cruz Biotechnology, Dallas, TX, USA) monoclonal antibodies. After incubation with appropriate peroxidase-conjugated anti-IgG (Amersham Biosciences, Milan, Italy), membranes were revealed using the ECL Chemiluminescent Substrate (ThermoFisher Scientific, Waltham, MA, USA).

### ELISA for IgG detection

Serial dilution of exosomes (from 20 × 10^9^ to 2.5 × 10^9^) were seeded in 96 well-plates (Nunc MaxiSorp, Milan, Italy) in 100 µl/well of carbonate buffer (pH 9,6) and incubated overnight at 4 °C. After three washes with PBS, 100 µl/well of blocking solution (PBS containing 0.5% BSA) was added at room temperature for 1 h. After three washes with PBS, 4 µg/ml of a goat anti-mouse IgG HRP (Amersham Biosciences, Milan, Italy) were added to each well and incubated for 1 h at 37 °C. After the final three washes with PBS, the reaction was developed with Blue POD for 15 min (Roche Applied Science, Milan, Italy), blocked with 4 N H2SO4 stop solution, and optical densities were recorded at 450 nm.

### Lymphocytes stimulation and intracellular staining

Peripheral blood mononuclear cells were isolated from whole blood by density gradient centrifugation using standard procedures (Ficoll-Paque Plus, GE Healthcare, Chicago, IL, USA). PBMC were plated at 0.5 × 10^6^ cells for well and cultured in the presence of decreasing dilutions (20, 10, 5, and 2,5 × 10^9^) of OKT3 exosome preparation, OKT3 supernatant and OKT3 supernatant without exosomes. As positive controls, anti-CD3 antibody (OKT3 clone, Miltenyi Biotec, Bergisch Gladbach, Germany) was added to the cells at 1 µg/ml in a soluble state or plate bound. As negative control decreasing dilutions of P3X63 exosome preparation were used. In all conditions, 10 υg/ml of Brefeldin A (Sigma Aldrich, Saint Louis, MO, USA) was added. After 7 h, PBMC were washed twice and labelled superficially with the following anti-human antibodies with the addition of LIVE/DEAD Fixable Aqua Dead Cell Stain (ThermoFisher, Waltham, MA, USA): CD3 (Beckman Coulter, Indianapolis, IN, USA), CD4 (Becton Dickinson, Franklin Lakes, NJ, USA), CD8 (Milteniy, Bergisch Gladbach, Germany) conjugated, respectively, with Pe-Cy7, Brilliant Violet 605 and APC-Vio770. The cells were then washed, fixed (0.5% formaldehyde) and permeabilized with 0.5% of Saponin solution for the intracellular staining. PBMC were labelled intracellularly with the following anti-human cytokines antibodies: IL2 (PE-dazzle-594 conjugated, Biolegend, San Diego, CA, USA), IFN-γ (Violet 450 conjugated, Becton Dickinson, Franklin Lakes, NJ, USA) and TNF-α (Fitc conjugated Milteniy, Bergisch Gladbach, Germany).

### Flow cytometry

Stained cells were acquired on a CytoFLEX flow cytometer (Beckman Coulter, Indianapolis, IN, USA), equipped with three lasers, and able to measure up to 15 parameters simultaneously on each cell.

For each sample, approximately 300,000 lymphocytes were selected based on scatter parameters, and the analysis was conducted after the exclusion of dead cells and coincident events.

The data was compensated and analysed using FlowJo v10.5 (TreeStar, Ashland, OR, USA).

## Results

### Characterisation of exosomes

OKT3 is a hybridoma cell line derived from the fusion of mouse B cell immunised for CD3, and P3X63 (P3x63Ag8.U1) myeloma cell line to generate a large amount of cells expressing identically IgG2a antibodies directed against the CD3 antigen of T Cells. OKT3 hybridoma cells already express the primary antibody, therefore it was necessary only the incubation with the goat anti IgG secondary antibody, directly conjugated to a fluorochrome, to detect the signal. We first checked the Ig expression in OKT3 cell line through Immunofluorescence Analysis (Supplementary Figure S1). OKT3 cell line had a strong plasma membrane-associated expression of total IgG compared to P3X63, our negative control, that did not show any immunoglobulins-related positivity (Supplementary Figure S1). Based on previous reports showing immunoglobulin expression on exosomes purified from either cell line supernatant or body fluids^32^^,^^38^^,^^40^^,^^41^, we preliminary evaluated the presence of exosomes in the supernatant of the OKT3 hybridoma cell line (IgG2a positive), or P3X63 (IgG2a negative) or human primary macrophages (negative control) through nanoparticle tracking analysis (NTA)^49^^,^^50^, confirming the presence of EVs with size within the typical range of nanovesicles/exosomes (Supplementary Figure S2).

**Figure 1.**
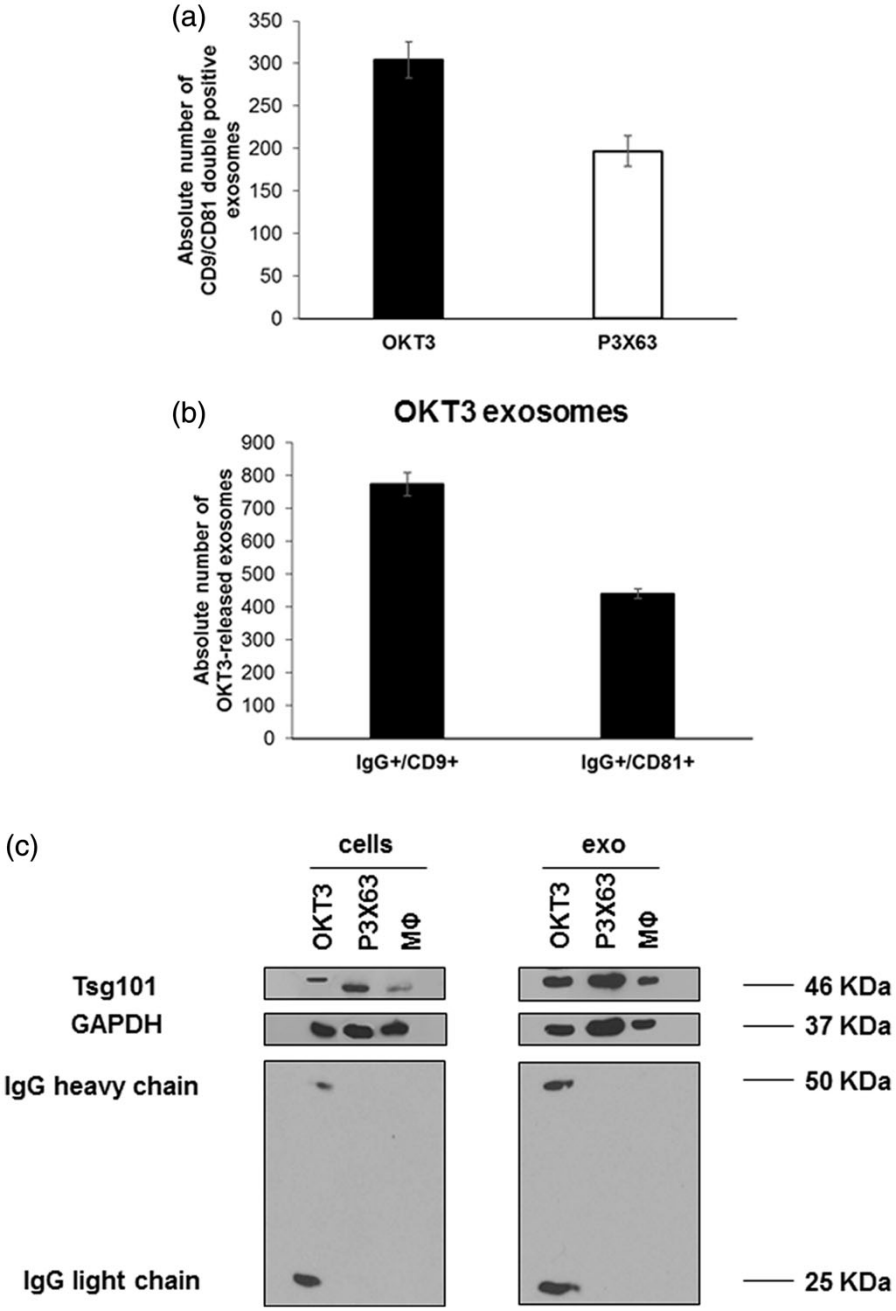
Characterisation of exosomes samples isolated from OKT3, P3X63 by nanoscale flow cytometry and western blot analysis. (a) Analysis of double positive exosomes for CD9 and CD81, typical exosomal markers, isolated from OKT3 and P3X63 cell lines by Nanoscale Flow Cytometry. Data are expressed as mean ± SD. (b) Analysis of IgG/CD9 and IgG/CD81 positive exosomes isolated from OKT3 cell culture medium by Nanoscale Flow Cytometry. Data are expressed as mean ± standard deviation. (c) Evaluation of immunoglobulins (heavy and light chains) and Tsg101 (typical exosomes marker) expression, in OKT3, P3X63 and human primary macrophages cells and exosomes samples by western blot analysis.

To provide more specific information on EVs released by OKT3 hybridoma cell line, we characterised our samples for the expression of typical exosomal markers, (i.e. CD9, CD81 and Tsg101), using two different techniques: nanoscale flow cytometry for surface markers CD9 and CD81 and western blot analysis for the expression of cytosolic protein Tsg101. The results of nanoscale flow cytometry showed that both OKT3 hybridoma and P3X63 cell lines were positive for CD9 and CD81 in the typical size range of exosomes (i.e. 110–180 nm) (Figure 1(a)). Moreover, western blot analysis showed that both exosomes and cells expressed fairly detectable Tsg101 (Figure 1(c)).

### Immunoglobulin expression in OKT3 exosomes samples

In order to evaluate the simultaneous expression of immunoglobulins and the exosomal markers, in EVs obtained from OKT3 hybridoma and P3X63 cell lines, samples were incubated with both anti-CD9 and CD81 mAbs and a goat anti-mouse antibody. The results analysed by nanoscale flow cytometry showed that the double IgG/CD9 and IgG/CD81 positive exosomes were exclusively detectable in samples obtained by OKT3 hybridoma (Figure 1(b)), while samples from the control hybridoma P3X63 exclusively expressed the exosomal markers (data not shown), thus supporting the expression of IgG in exosomes derived from OKT3 cells. To further confirm these results, a western blot analysis aimed at evaluating the presence of IgG bands in the EVs samples was performed. The results showed that bands of both heavy and light IgG chains were detectable in extracts of both cells and exosomes samples from OKT3 hybridoma (Figure 1(c)), while both cells and exosomes from P3X63 cell line and human primary macrophages were immunoglobulin negative.

### Purification of exo OKT3 preparation through a sucrose density gradient

**Figure 2.**
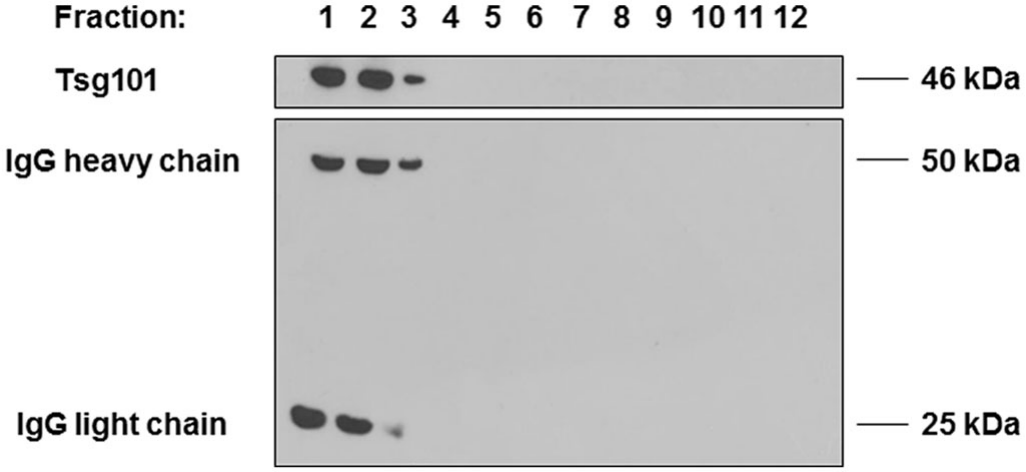
Characterisation of immunoglobulins and Tsg101 expression in OKT3 exosomes after purification by sucrose gradient. After isolation from OKT3 cells culture medium, exosomes were loaded on 30%/D2O sucrose gradient and subsequently analysed by Western Blot. Heavy and light chain of immunoglobulins were detectable in the same fractions of Tsg101.

To eliminate contaminating material, such as protein aggregates and nucleosomal fragments released by apoptotic bodies, and to investigate the specific presence of immunoglobulins in exosomal preparations, we performed a sucrose density gradient analysis^48^^,^^51^^,^^52^. After isolation from OKT3 cell culture medium, exosomes samples were loaded on 30%/D2O sucrose gradient and centrifuged again at 110,000 g. After that, the density fractions were analysed by western blot to identify the expression of immunoglobulins and Tsg101, a typical exosomal marker. As shown in Figure 2, fractions 1, 2 and 3 revealed the expression of IgG heavy and light chains together with the presence of the exosomal marker Tsg101, thus showing that the immunoglobulin bands were fairly detectable in the same fractions where the Tsg101 exosomal marker was detectable, thus showing that the IgG heavy and light chains were associated to the exosomes released by the OKT3 hybridoma.

### Immunoglobulin detection is related to exosomes number

**Figure 3.**
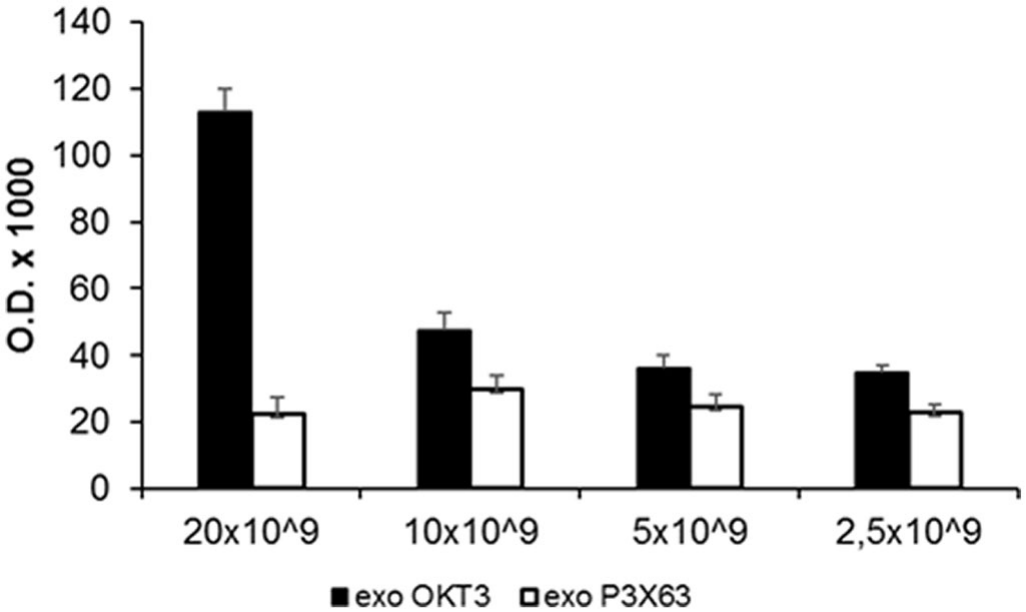
Characterisation of immunoglobulins in different amount of OKT3 exosomes by ELISA. Different amount of OKT3 and P3X63 exosomes (20-10-5 and 2.5 × 10^9^) were directly seeded to Nunc MaxiSorp plates and then incubated with anti-mouse antibody to evaluate immunoglobulins expression related to exosomes number. Optical densities are expressed as mean ± SD.

The previous set of experiments has shown that exosome samples obtained from the OKT3 hybridoma contained both the heavy and the light chains of immunoglobulins. However, we did not know whether the immunoglobulins were specifically expressed on exosomes. Thus, we performed a new series of experiments aimed at demonstrating a direct relationship between exosomes and the monoclonal antibodies released by the OKT3 hybridoma. To this purpose, we performed an ELISA assay in order to detect and quantify the immunoglobulins on the exosomes released by the OKT3 hybridoma. Based on the natural capacity of exosomes to bind to Nunc plates, different amounts of exosomes (from 20 × 10^9^ to 2.5 × 10^9^) were seeded on plastic plates and incubated with anti-mouse-HRP antibody to detect immunoglobulins. As shown in Figure 3, OKT3 exosomes showed an expression of immunoglobulin depending on exosomes amount, while the P3X63 exosomes did not show positivity for the immunoglobulins. This experiment showed that immunoglobulins were exclusively detectable in exosome isolated from OKT3 hybridoma cell line and that the expression was related to the exosomes number.

### OKT3 exosomes stimulate lymphocyte cytokine production

The previous set of experiments has shown that exosomes released by the OKT3 hybridoma specifically express heavy and light chains of immunoglobulins and these immunoglobulins were expressed on exosomes. However, we did not know whether the IgG-bearing exosomes could exert a specific function related to the OKT3 monoclonal antibody. Thus, we performed a new series of experiments aimed at demonstrating the specific functionality of the OKT3 exosomes. To test the functional properties of the antibodies associated with exosomes, we performed a series of new experiments in which exosomes purified from supernatants of the OKT3 and P3X63 cell lines were added to human PBMC cultures and the IFN-γ, IL-2 and TNF-β production assayed by intracellular staining. To this purpose, we used the following experimental conditions: (1) different concentrations of the exosome preparations deriving from the OKT3 hybridoma; (2) the exosome-free supernatant deriving from the exosome preparation (that should include the soluble antibodies not associated with exosomes); (3) the total supernatant of the hybridoma containing both the exosomes and the soluble antibodies.

**Figure 4.**
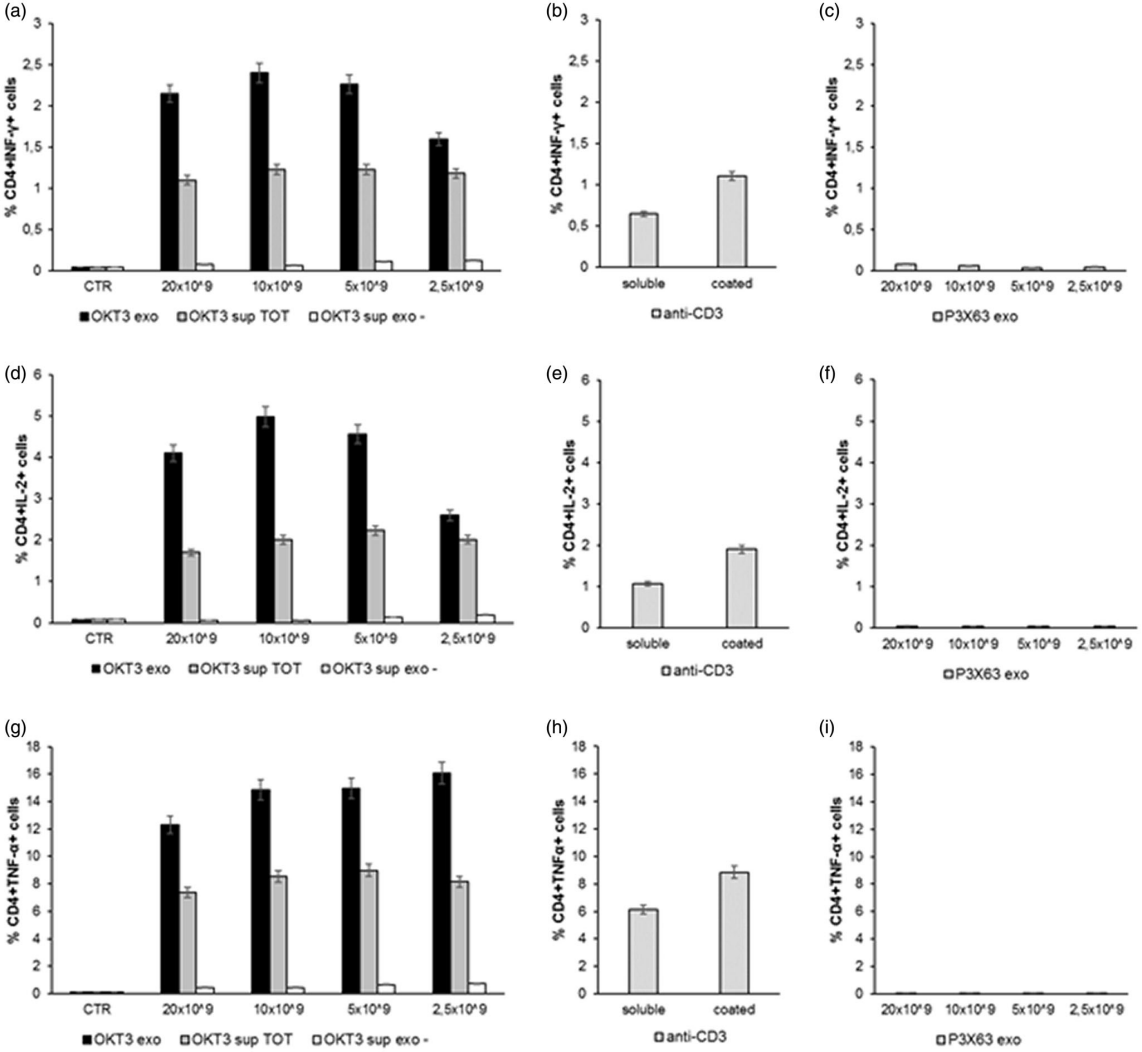
Analysis of cytokines production by Lymphocytes CD4+ after incubation with exosomes. Human PBMC were stimulated with OKT3 exosomes at different concentration (20-10-5 and 2.5 × 10^9^); supernatant of hybridoma cell line (OKT3 sup TOT); supernatant of exosomes (OKT3 sup exo-); anti-CD3 soluble and plate bound (1υg/ml); P3X63 exosomes (20-10-5 and 2.5 × 10^9^). After 7 hours the cells were stained for the intracellular cytokines detection. Intracellular staining of CD4+ Lymphocytes for IFN-γ (a, b and c), IL-2 (d, e and f) and TNF-α (g, h and i). Data are expressed as mean ± SD.

**Figure 5.**
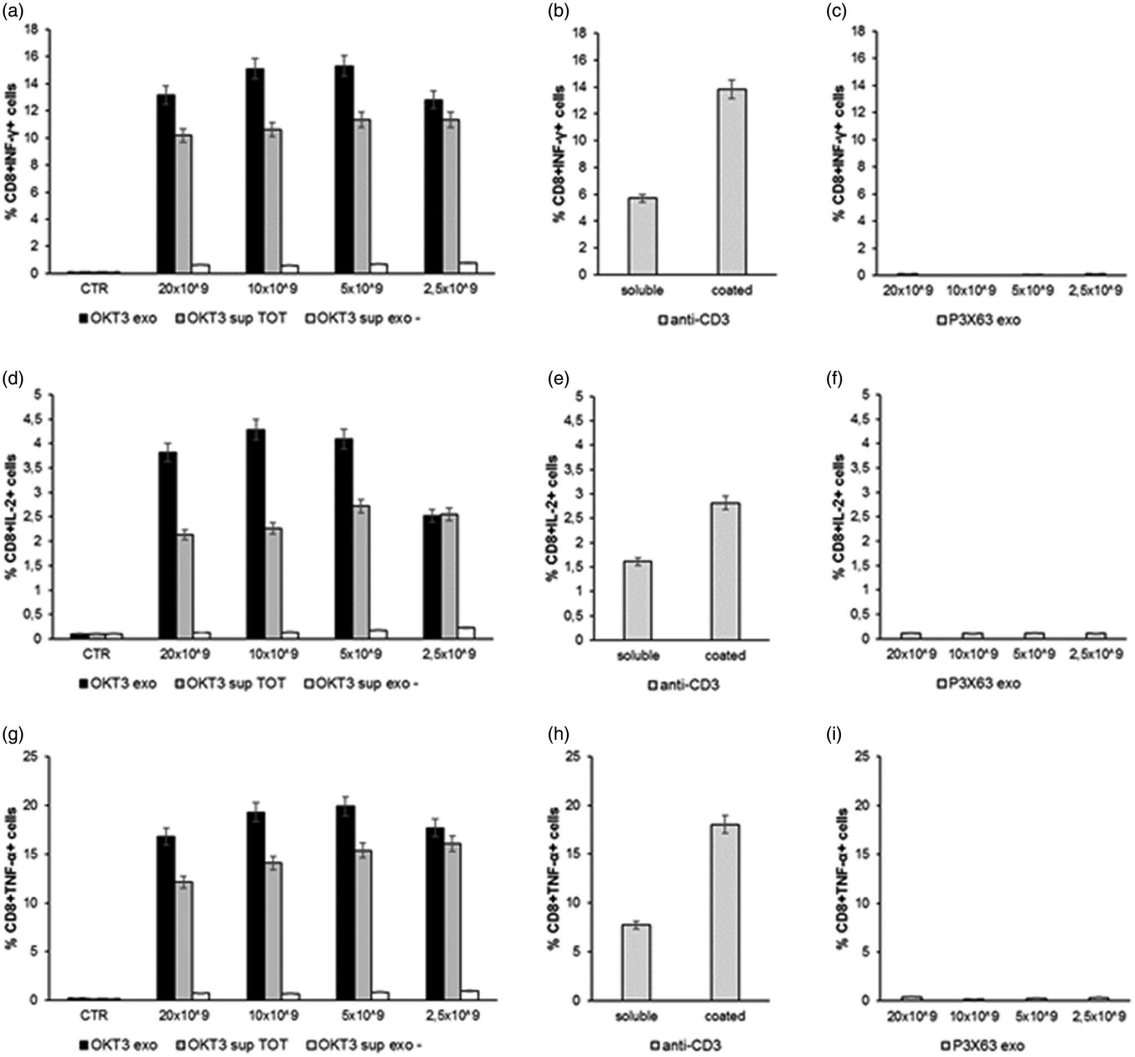
Analysis of cytokines production by lymphocytes CD8+ after incubation with exosomes. Human PBMC were stimulated with OKT3 exosomes at different concentration (20-10-5 and 2.5 × 10^9^); supernatant of hybridoma cell line (OKT3 sup TOT); supernatant of exosomes (OKT3 sup exo-); P3X63 exosomes (20-10-5 and 2.5 × 10^9^); anti-CD3 soluble and plate bound (1υg/ml); P3X63 exosomes (20-10-5 and 2.5 × 10^9^). After 7 h, the cells were stained for the intracellular cytokines detection. Intracellular staining of CD8+ Lymphocytes for IFN-γ (a, b and c), IL-2 (d, e and f) and TNF-α (g, h and i). Data are expressed as mean ± SD.

The results showed that the exosome preparations from the OKT3 hybridoma induced by far the highest cytokine production in both CD4 T (Figure 4) and CD8 T lymphocytes (Figure 5) cultures. The highest percentage of CD4+ or CD8+/cytokine + cells was induced at the concentration of 10 × 10^9^ exosomes, beginning to decrease at the concentration of 2.5 × 10^9^ (Figures 4 and 5(a,d,g)). Of interest, the increase in cytokine production was higher when lymphocytes were stimulated with the exosomes-associated anti-CD3 than the stimulation with the purified anti-CD3 antibody (OKT3 clone), at the standard concentration of 1 µg/ml, either plate-bound or soluble (Figures 4 and 5(b,e,h)). Exosomes isolated from the control P3X63 cell line did not show to stimulate cytokines production by either CD4+ or CD8+ T lymphocytes (Figures 4 and 5(c,fi)).

## Discussion

Antibody-based therapies are gaining increasing interest in medicine and they are paving the way for modern immunotherapy. Monoclonal antibodies are used against various diseases, including cancer and autoimmune diseases. Monoclonal antibodies-mediated therapies are an example of direct targeting therapy and they aim at curing diseases by binding disease-associated epitopes, recognised by monoclonal antibodies. However, despite the promising results, clinical application is limited due to (i) the high costs hardly sustainable for the public healthcare systems, (ii) the critical level of toxicity^53^, frequently leading to the interruption of therapies and (iii) unfortunately a low level of efficacy probably due to an unexpected low specificity of the antibodies. More research is needed to identify better formulations allowing a better clinical efficacy of therapeutic antibodies, together with a reduced level of systemic toxicity.

This study was triggered by some recent findings showing that exosomes from immune cells express immunoglobulins^54^, and our personal observation that exosomes purified from the plasma of experimental animals contain both light and heavy chains of immunoglobulins^38^, that are always undetectable in cell culture supernatants but those derived from immune cells. On the basis of these preliminary observations, we hypothesised that one major and natural way of antibody production could be through exosome release. To this purpose, we used the OKT3 hybridoma expressing an IgG2a antibody recognising the CD3 of T cells. This antibody (Muromab-CD3) is commonly used to prevent rejection of organ transplantation. The results showed that the OKT3 hybridoma released typical exosomes, in terms of both distribution and size, that expressed the typical markers such as CD9, CD81 and Tsg101. Contextually to the characterisation of the exosomes, we verified the expression of immunoglobulins on exosomes derived by OKT3 hybridoma by Western Blot Analysis and nanoscale flow cytometry: given the possible overlap of the CD9 and CD81 bands with the immunoglobulin light chain, we decided to verify the co-expression of Ig and Tsg101 by western blot and of Ig with CD9 and CD81 by cytometry. The results showed the ability of the hybridoma to release exosomes expressing antibodies, that while reported in cultures of other cell^35^^,^^38–42^^,^^55^, represents definitively a novelty. The results obtained with Western Blot Analysis and nanoscale flow cytometry were supported by the immunocapture-based ELISA showing the expression of immunoglobulins in exosomal preparations derived from the OKT3 hybridoma, that increased along with the increase of the exosomes seeded on the plastic plates.

In a further set of experiments, we showed that exosomes from OKT3 hybridoma were able to induce marked cytokine release by both CD4+ and CD8+ T cells, related to a specific interaction of the exosome-related OKT3 antibody with the T-cell receptor. This is a key new discovery, inasmuch as in the past only an aspecific EVs-mediated T cells activation was reported^29^^,^^35^^,^^56^^,^^57^.

All in all our data show that: (i) exosome released by an hybridoma express a monoclonal antibody used in immunotherapy (Muromab-CD3); (ii) the supernatant of the purified exosomes does not contain functional antibodies, suggesting that the great majority of the monoclonal antibody is associated to the exosomes; (iii) that the monoclonal antibody associated to the hybridoma-released exosomes were fully functional in inducing a potent cytokine release by primary T cells, either CD4 or CD8; (iv) the CD3-bearing exosomes were more effective than the soluble antibody, in turn suggesting a potential and more effective role of immunoglobulin-expressing exosomes as compared to soluble monoclonal antibodies in all immunotherapeutic approaches. This appears conceivable in as much as it has been widely documented that some antibodies are not effective in their soluble form as compared to the immobilisation on a surface^21–23^. Moreover, our results support a new deal for the formulation of therapeutic antibodies being probably the release of exosome with Igs (exo-Ig) anchored to their membrane a natural event and in fact our results have shown that exo-Ig are much more effective than soluble Igs. Even more, given the crucial role of these vesicles in intercellular communication, the exo-Ig could improve the limited capacity of soluble immunoglobulins to spread from the bloodstream, across vascular endothelial cells and then to the tissues^53^^,^^58^^,^^59^. Furthermore, when immunoglobulins reach their target and are internalised, they are subjected to rapid degradation in lysosomes^58^^,^^60–62^, avoiding the damage due to the formation of the immune complex. Additionally, the exosomes are able to cross the blood-brain barrier, which still remains one of the limits of immunoglobulin that have to be conjugated to a specific carrier (e.g. liposomes)^63–65^ to become fully effective.

Lastly, our results are particularly relevant to the current COVID-19 pandemic. While vaccine research is currently undergoing, more readily available treatments including monoclonal antibodies for passive immunotherapy may be crucial to fighting against viral infection. The therapeutic potential of monoclonal antibodies has been well recognised in the treatment of SARS-CoV and MERS-CoV. Exosome-associated antibody delivers efficient and precise therapies and contributes to improving our response to fighting these viruses.

In conclusion, the results of this study open a new path for the production of therapeutic antibodies linked to exosomes. This could be obtained by purifying exosomes released by hybridomas or by using exosomes released by primary normal cells to be linked to therapeutic antibodies.

## Supplementary Material

Supplemental Material
