# Supplementary material for: Nanovesicles released by OKT3 hybridoma express fully active antibodies
Source: J Enzyme Inhib Med Chem. 2021 Jan 6;36(1):175–82. doi: 10.1080/14756366.2020.1852401 (PMC7801098; doi:10.1080/14756366.2020.1852401)
Supplement: Supplemental Material [file IENZ_A_1852401_SM7236.pdf]

Supplemental material

NANOVESICLES RELEASED BY OKT3 HYBRIDOMA EXPRESS FULLY ACTIVE ANTIBODIES

Supplementary Figure 1

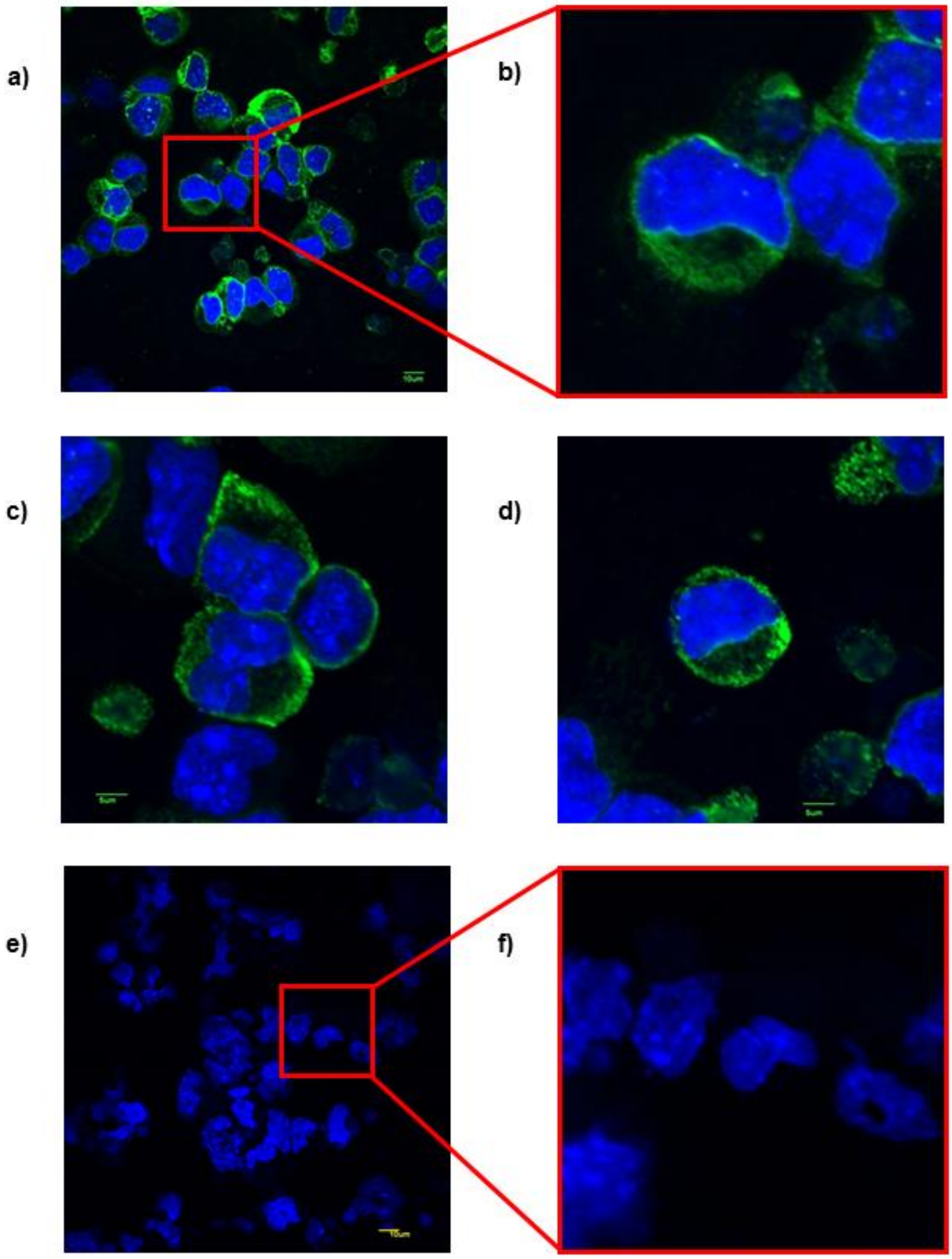

Supplementary Figure 1. Laser scanning confocal microscopy (LSCM) of immunoglobulins expression in OKT3 and P3X63 cell lines

OKT3 cell line showed a great expression of membrane-bound immunoglobulins (**a**, **b**, **c** and **d**), while P3X63, our negative control, did not show any expression of immunoglobulins (**e** and **f**)

Supplementary Figure 2

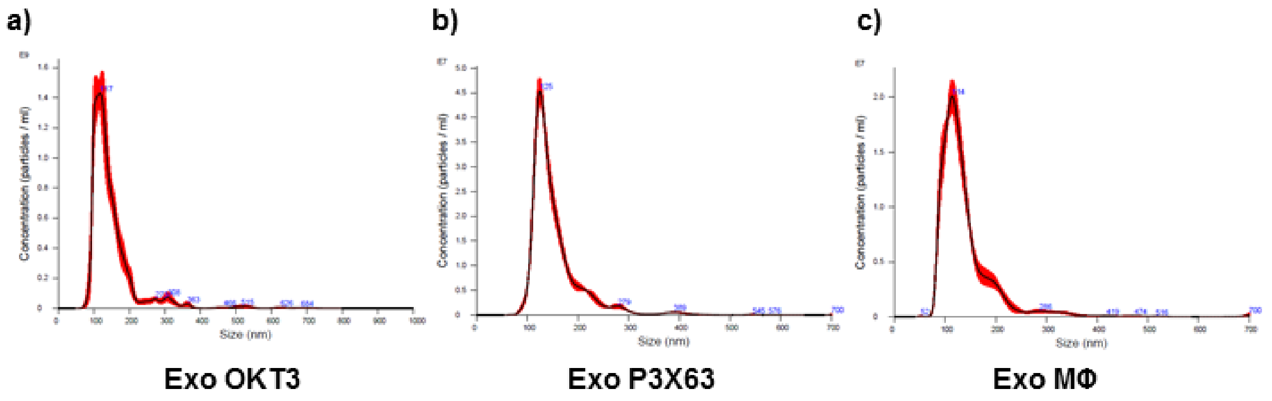

Supplementary Figure 2. Characterization of exosomes samples isolated from OKT3, P3X63 and human primary macrophages by Nanoparticle Tracking Analysis

Analysis of size and distribution of exosome derived from OKT3 hybridoma cell line (**a**), P3X63 (**b**) and Human Primary Macrophages (**c**) by Nanoparticle Tracking Analysis
